# Cardiovascular impairment in Shiga-toxin-2-induced experimental hemolytic–uremic syndrome: a pilot study

**DOI:** 10.3389/fimmu.2023.1252818

**Published:** 2023-09-22

**Authors:** Charles Neu, Bianka Wissuwa, Christoph Thiemermann, Sina M. Coldewey

**Affiliations:** ^1^ Department of Anesthesiology and Intensive Care Medicine, Jena University Hospital, Jena, Germany; ^2^ Septomics Research Center, Jena University Hospital, Jena, Germany; ^3^ Center for Sepsis Control and Care, Jena University Hospital, Jena, Germany; ^4^ William Harvey Research Institute, Barts and the London School of Medicine and Dentistry, Queen Mary University of London, London, United Kingdom

**Keywords:** hemolytic-uremic syndrome, Shiga toxin, acute kidney injury, echocardiography, murine model, cardiomyopathy

## Abstract

**Introduction:**

Hemolytic–uremic syndrome (HUS) can occur as a systemic complication of infection with Shiga toxin (Stx)-producing Escherichia coli (STEC). Most well-known aspects of the pathophysiology are secondary to microthrombotic kidney disease including hemolytic anemia and thrombocytopenia. However, extrarenal manifestations, such as cardiac impairment, have also been reported. We have investigated whether these cardiac abnormalities can be reproduced in a murine animal model, in which administration of Stx, the main virulence factor of STEC, is used to induce HUS.

**Methods:**

Mice received either one high or multiple low doses of Stx to simulate the (clinically well-known) different disease courses. Cardiac function was evaluated by echocardiography and analyses of biomarkers in the plasma (troponin I and brain natriuretic peptide).

**Results:**

All Stx-challenged mice showed reduced cardiac output and depletion of intravascular volume indicated by a reduced end-diastolic volume and a higher hematocrit. Some mice exhibited myocardial injury (measured as increases in cTNI levels). A subset of mice challenged with either dosage regimen showed hyperkalemia with typical electrocardiographic abnormalities.

**Discussion:**

Myocardial injury, intravascular volume depletion, reduced cardiac output, and arrhythmias as a consequence of hyperkalemia may be prognosis-relevant disease manifestations of HUS, the significance of which should be further investigated in future preclinical and clinical studies.

## 1 Introduction

Diarrhea-positive hemolytic–uremic syndrome (HUS) is a microthrombotic renal condition often caused by an infection with enterohemorrhagic *Escherichia coli* (EHEC). It is the most frequent cause of acute kidney injury (AKI) in children (1); further symptoms include thrombocytopenia and acute hemolytic anemia. As key virulence factors, EHEC secrete ribosome-inactivating Shiga toxins (Stx, type 1 and 2), which are often responsible for organ failure. Epidemiological data revealed that Stx2 is more frequently associated with the development of hemorrhagic colitis in HUS (2). Aside from renal and hematological symptoms, neurological and cardiac complications are known (3). Several case reports describe an impairment of cardiac function in patients with HUS. Specifically, patients with HUS exhibited a reduced left-ventricular systolic function and an increase in plasma troponin I—the latter of which is a well-known surrogate (bio)marker of myocardial injury (4–6)—as well as the need for cardiopulmonary resuscitation via cardiopulmonary bypass (4). Troponins are sensitive biochemical markers that are released into the bloodstream during myocardial injury. Cardiac troponin I (cTNI), one of three isoforms, is a specific serum marker of cardiac injury. Increases of cTNI can occur prior to and following severe myocardial dysfunction in HUS (5); however, as troponins are excreted via the kidney, their concentrations may also increase during AKI in the absence of myocardial damage [reviewed in (7)]. In some cases, the cause for the observed cardiac events during HUS was suspected to be ischemic in nature (8). Accordingly, case series of autopsied patients with diarrhea-associated HUS showed microthrombi formation in myocardial tissue (9), myocardial infarction (10), or pericardial purpura (11). The pathophysiological mechanisms leading to severe cardiac complications in some patients but not in others have not been fully understood. Furthermore, no specific treatment options exist for this oftentimes fatal extrarenal manifestation of HUS. Likewise, in atypical HUS (aHUS)—caused by mutations in the complement system in the absence of an EHEC infection—cases of cardiac dysfunction have also been reported with similar clinical presentation (12–14). In aHUS, however, immunomodulatory therapy (15) led to a reversal of cardiac impairment (12).

We have previously established and characterized two murine models of HUS based on the intravenous injection of Stx with two distinct courses of disease progression: an acute model of up to 3 days and a subacute model over the course of 1 week (16). In order to investigate the impact of Stx on the development of cardiac dysfunction in experimental HUS, we expanded our previously published models in a proof-of-concept manner, by performing echocardiography and measuring cardiac markers in the plasma of mice in both the acute and subacute model of HUS to better understand the potential and limitations of these models for the assessment of extrarenal cardiac manifestations of HUS in a pilot study. The echocardiography was complemented by hematological analysis and plasma measurements of kidney function as in previous experiments (16).

## 2 Materials and methods

### 2.1 Mice (*Mus musculus*)

Male wild-type C57BL/6J mice [aged 10–12 weeks, 20 to 30 g body weight (BW)] were randomly assigned to one of the two models, acute or subacute and treatment groups Stx or sham (acute Stx *n* = 11, all other groups *n* = 10), by a computer-generated list. Mice were kept under standardized laboratory conditions enriched with shredded paper and paper rolls, and received standard rodent chow and water *ad libitum*.

Experiments were performed in accordance with approved guidelines and the German law for animal protection. The animal experiment proposal was approved by the Thuringian State Office for Consumer Protection, Bad Langensalza, Germany (registration number 02-073/16).

### 2.2 Induction of HUS

HUS was induced by either a single i.v. injection of Stx (300 ng/kg BW in 5 mL/kg NaCl 0.9%; acute model) or two i.v. injections of Stx (25 ng/kg BW in 5 mL/kg BW NaCl 0.9% each; subacute model) on days 0 and 3. Sham mice received 5 mL/kg NaCl 0.9% at the corresponding time points. To account for the exacerbation of the condition of mice during anesthesia for echocardiography, the experimental protocol was shortened by 1 (acute model) and 2 days (subacute model), respectively, but remained otherwise unchanged (16). Stx was obtained from the same stock as previous experiments (16). Investigators were blinded for the treatment allocation of mice during the experiment. All mice received a volume resuscitation of 0.5 mL of Ringer’s lactate solution s.c. twice daily in the acute model and 0.8 mL of Ringer’s lactate solution s.c. three times a day in the subacute model. Clinical presentation of animals was assessed three times a day throughout the experiment using a scoring system previously described (16). The HUS score categorizes the following degrees of severity: 1 = no signs of illness, 2 = low-grade, 3 = mid-grade, and 4 = high-grade disease. Pre-defined criteria were assessed to determine whether termination of the experiment was necessary employing humane endpoints. Experiments were terminated on day 2 in the acute model and on day 5 in the subacute model of HUS. Blood samples were obtained by cardiac puncture in deep anesthesia during termination of the experiment.

### 2.3 Echocardiography

Echocardiography was performed with the Vevo 3100 imaging system (FUJIFILM VisualSonics, Inc., Toronto, ON, Canada) using a 32–55 MHz MX550D transducer (FUJIFILM VisualSonics, Inc.). Ultrasound analyses were performed at three time points in both models of HUS. T0 corresponds to the time point before induction of HUS to measure basic values in a healthy condition. T1 and T2 represent day 1 and day 2 in the acute model, and day 3 and day 5 in the subacute model, respectively. Anesthesia was induced with 3% isoflurane (CP-Pharma, Burgdorf, Germany) and maintained at 1%–1.5%. Ultrasound images were recorded after a stabilization period of approximately 10 min. ECG monitoring was performed during the entire anesthesia using four paw contact electrodes of the Vevo 3100. Body temperature was measured by a rectal thermometer during anesthesia. To assess cardiac function, LV B-mode and M-mode images of parasternal long axis (PSLAX) and short axis (PSAX) as well as B-mode and pulsed-wave (PW) Doppler mode images of the pulmonary artery were recorded. Analyses of ultrasound images were conducted by two independent researchers in a blinded manner using the Vevo LAB 3.2.0 software (FUJIFILM VisualSonics, Inc.). EF and EDV were calculated by manual LV tracing measurement in PSLAX B-mode. Cardiac output (CO) was calculated from the diameter of the pulmonary artery in B-mode and manual velocity time integral (VTI) measurement in PW Doppler mode.

### 2.4 Hematology and plasma analyses

Whole blood analysis was performed using the scil Vet abc Plus^+^ hematology analyzer (scil animal care company GmbH, Viernheim, Germany) to quantify red blood cell count, platelet count, hematocrit, hemoglobin, and mean corpuscular volume. Plasma was prepared by centrifugation of heparinized whole blood for 10 min at 3000 g. Creatinine, urea, sodium, and potassium were quantified in plasma samples with the ARCHITECT™ *ci*16200 System (Abbott, Chicago, IL, USA). NGAL was quantified in plasma samples by ELISA (Cat. No. 443707; BioLegend^®^, Inc., San Diego, CA, USA). cTnI levels were determined in plasma samples by ELISA (CTNI-1-HSP; Life Diagnostics, Inc., West Chester, PA, USA). Quantification of brain natriuretic peptide (BNP) was performed by an enzyme immunoassay (RAB0386; Sigma-Aldrich, St. Louis, MO, USA). In some cases, not all measurements were possible due to too little plasma volumes.

### 2.5 Statistical analysis

Data are displayed as mean ± standard deviation (SD) of *n* observations representing the number of animals. Data were pooled from two replicate experiments. Statistical analysis was performed using GraphPad Prism 7.05 (GraphPad Software, Inc., San Diego, CA, USA). Data were analyzed by Mann–Whitney *U* test and two-way repeated measures ANOVA followed by Bonferroni’s multiple comparison test as indicated in the respective figure legend (*p < 0.05, **p < 0.01, ***p < 0.001, ****p < 0.0001).

## 3 Results

### 3.1 The effect of a single high-dose challenge with Stx on systemic disease and kidney dysfunction and injury

**Figure 1:**
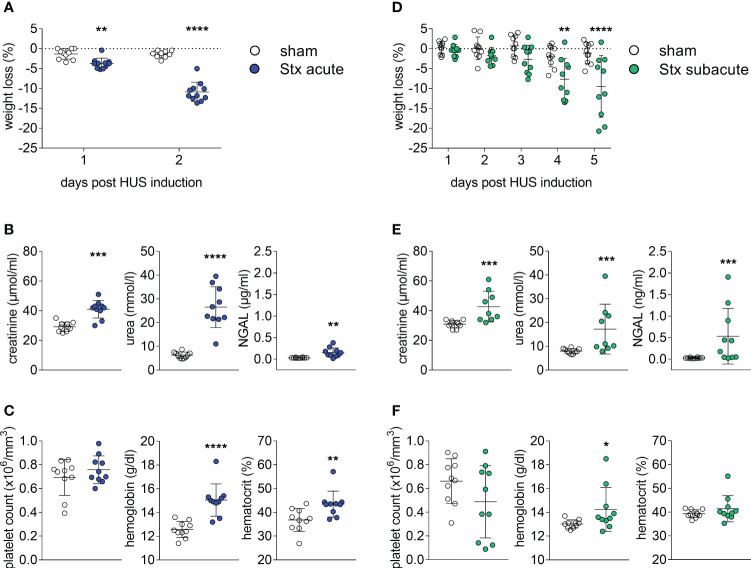
Clinical presentation of hemolytic–uremic syndrome (HUS) in mice exposed to different Shiga toxin (Stx) application regimes. **(A–C)** C57BL/6J mice received either a single high dose of 300 ng/kg Stx (Stx acute) or vehicle (sham). Blood was taken at the end of the experiment, 2 days after the induction of HUS **(B, C)**. **(A)** Weight loss percentage of body weight (sham *n* = 10, Stx acute *n* = 11). **(B)** Plasma levels of creatinine and urea (sham *n* = 10, Stx acute *n* = 9) and neutrophil gelatinase-associated lipocalin (NGAL) (sham *n* = 10, Stx acute *n* = 10). **(C)** Platelet count, hemoglobin, and hematocrit (sham *n* = 10, Stx acute *n* = 10) **(D–F)**. Mice received either two lower doses of 25 ng/kg Stx (Stx subacute) spaced 72 h apart or vehicle (sham). Blood was taken at end of the experiment, five days after the induction of HUS **(E, F)**. **(D)** Weight loss percentage of body weight (sham *n* = 10, Stx subacute *n* = 10). **(E)** Plasma levels of creatinine and urea (sham *n* = 10, Stx subacute *n* = 9) and NGAL (sham *n* = 10, Stx subacute *n* = 10). Platelet count, hemoglobin, and hematocrit (sham *n* = 10, Stx subacute *n* = 10). **(A–F)** Data are presented as scatter dot plot with mean ± SD for *n* number of observations, *p < 0.05, **p < 0.01, ***p < 0.001 and ****p < 0.0001 vs. the corresponding sham group (**A, D**: two-way repeated measures ANOVA, Bonferroni’s multiple comparison test; all other: Mann–Whitney *U* test).

All mice challenged with a single dose of 300 ng/kg Stx (acute model of HUS) developed significant weight loss after 2 days (**Figure 1A**). When compared with sham-treated mice, plasma concentrations of creatinine, urea, and neutrophil gelatinase-associated lipocalin (NGAL) were increased 2 days after administration of Stx (**Figure 1B**), indicating acute kidney dysfunction and injury. The disease severity score, i.e., the humane endpoint of this experiment that was implemented as part of the termination criteria (s. HUS score, **Supplementary Figure 1A**), was significantly increased 48 h after administration of Stx, while sham-treated mice developed no signs of disease. Hematological analysis (**Figure 1C**) revealed significantly higher levels of hemoglobin and hematocrit in Stx-treated mice than in sham-treated mice. These results were in accordance with our previous work (Dennhardt et al., 2018).

### 3.2 The effect of repeated administrations of lower doses of Stx on systemic disease and kidney dysfunction and injury

All mice challenged with two doses of 25 ng/kg of Stx (subacute model of HUS) developed significant weight loss after 4 days (**Figure 1D**), which was more pronounced on the fifth and final day of the experiment. When compared with sham-treated mice, plasma concentrations of creatinine, urea, and NGAL (**Figure 1E**) were increased 5 days after administration of Stx, indicating acute kidney dysfunction and injury. The disease severity score (s. HUS score, **Supplementary Figure 1B**) was increased in a subgroup of mice 120 h after administration of Stx, while sham-treated mice showed no signs of disease. The hematological analysis (**Figure 1F**) revealed higher hemoglobin levels in Stx-treated mice when compared with sham. A subset of mice also developed thrombocytopenia.

### 3.3 The effect of a single high-dose challenge with Stx on cardiac function

**Figure 2:**
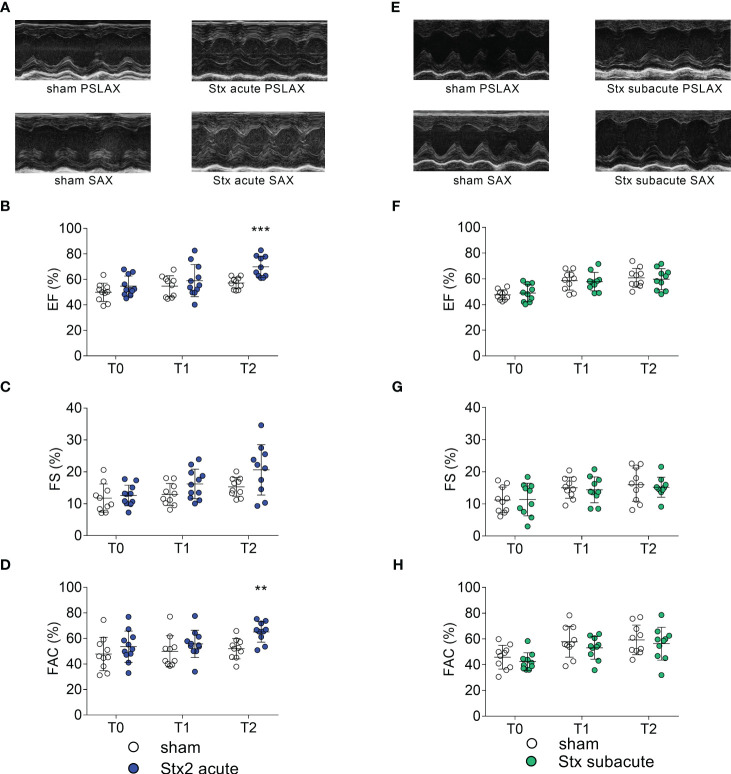
Left ventricular cardiac function in mice with hemolytic–uremic syndrome (HUS) exposed to different Shiga toxin (Stx) application regimes. **(A–D)** C57BL/6J mice received either a single high dose of 300 ng/kg of Stx (Stx acute, *n* = 11) or vehicle (sham *n* = 10). Echocardiography was performed before (T0, baseline) and 1 (T1) and 2 (T2) days after induction of HUS. **(A)** Representative parasternal long axis (PSLAX) and short axis (SAX) M-mode echocardiograms (0.5 s each) at T2. Left ventricular (LV) **(B)** ejection fraction (EF), **(C)** fractional shortening (FS), and **(D)** fractional area change **(FAC)** were analyzed to characterize LV cardiac function. **(E–H)** Mice received either two lower doses of 25 ng/kg Stx (Stx subacute *n* = 10) spaced 72 h apart or vehicle (sham *n* = 10). Echocardiography was performed before (T0, baseline) and 1 (T1) and 2 (T2) days approximately 10 min after induction of HUS. **(E)** Representative PSLAX and SAX M-mode echocardiograms (0.5 s each) at T2. LV **(F)** EF, **(G)** FS, and **(H)** FAC were analyzed to characterize LV cardiac function. **(B–D, F–H)** Data are presented as scatter dot plot with mean ± SD, **p < 0.01, ***p < 0.001 vs. the corresponding sham group (Mann–Whitney *U* test).

We analyzed left-ventricular function in the acute model of HUS by echocardiography before and after 1 and 2 days after the induction of HUS (**Figures 2A–D**). When compared with sham-treated mice, ejection fraction (EF) (**Figure 2B**) and fractional area change (FAC) were increased 2 days after administration of Stx, indicating increased left-ventricular contraction secondary to activation of the sympathetic nervous system (**Figure 2D**).

### 3.4 The effect of repeated administrations of lower doses of Stx on cardiac function

We analyzed left-ventricular function in the subacute model of HUS, before and after 3 and 5 days after induction of HUS (**Figures 2E–H**). When compared with sham-treated mice, echocardiographic analysis showed no significant changes in EF (**Figure 2F**), fractional shortening (FS, **Figure 2G**), and FAC (**Figure 2H**) at any time point.

### 3.5 The effect of a single high-dose challenge with Stx on cardiac function and hemodynamics

**Figure 3:**
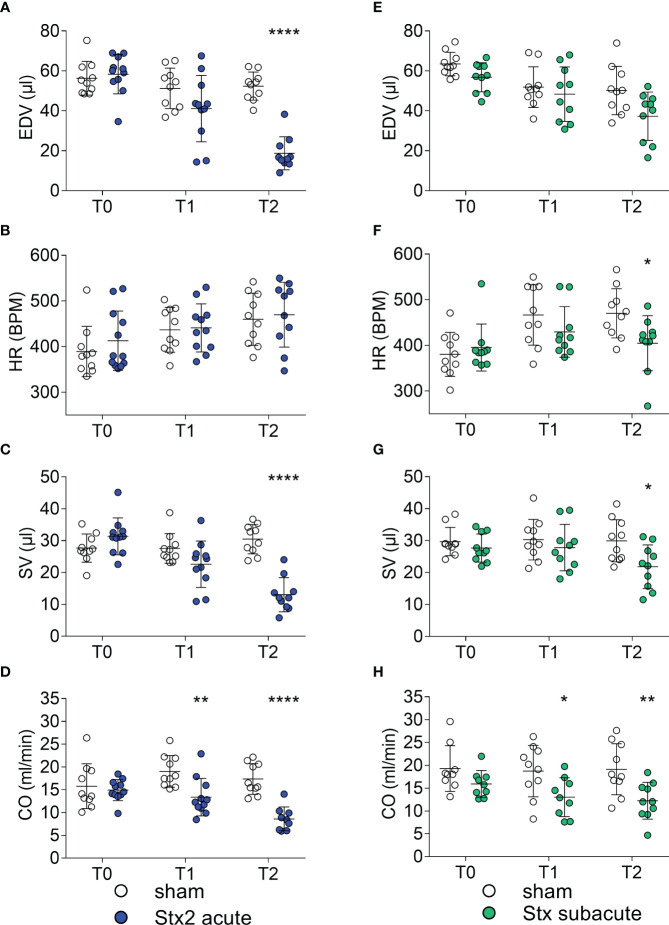
Hemodynamic parameters in mice with hemolytic–uremic syndrome (HUS) exposed to different Shiga toxin (Stx) application regimes. **(A–D)** C57BL/6J mice received either a single high dose of 300 ng/kg of Stx (Stx acute, *n* = 11) or vehicle (sham *n* = 10). Echocardiography was performed before (T0, baseline) and 1 (T1) and 2 (T2) days after induction of HUS. **(A)** End-diastolic volume (EDV), **(B)** heart rate (HR) in beats per minute (BPM) approximately 10 min after induction of anesthesia, **(C)** stroke volume (SV), and **(D)** cardiac output (CO) were analyzed to characterize hemodynamic changes. **(E–H)** Mice received either two lower doses of 25 ng/kg Stx (Stx subacute *n* = 10) spaced 72 h apart or vehicle (sham *n* = 10). Echocardiography was performed before (T0, baseline) and 1 (T1) and 2 (T2) days after induction of HUS. **(E)** EDV and **(F)** HR in BPM approximately 10 min after induction of anesthesia, **(G)** SV, and **(H)** CO were analyzed to characterize hemodynamic changes. **(A–H)** Data are presented as scatter dot plot with mean ± SD, *p < 0.05, **p < 0.01, ****p < 0.0001 vs. the corresponding sham group (Mann–Whitney *U* test).

We analyzed hemodynamically relevant parameters in the acute model of HUS by echocardiography before and after 1 and 2 days after the induction of HUS (**Figures 3A–D**). When compared with sham-treated mice, end-diastolic volume (EDV) was reduced 2 days after the induction of HUS (**Figure 3A**). There were no differences in heart rates (HR) between the groups measured after induction of anesthesia (**Figure 3B**). Stroke volume (SV) (**Figure 3C**) and CO (**Figure 3D**) were reduced 2 days after the induction of HUS, of which CO was also reduced 1 day after the induction of HUS (**Figure 3D**). There were no differences in heart rates between the groups measured after the induction of anesthesia. Taken together, the hemodynamic changes indicate intravascular volume depletion.

### 3.6 The effect of repeated administrations of lower doses of Stx on cardiac function and hemodynamics

We analyzed hemodynamically relevant parameters in the subacute model of HUS by echocardiography before and after 3 and 5 days after the induction of HUS (**Figures 3E–H**). When compared with sham-treated mice, EDV (**Figure 3E**), HR (**Figure 3F**), SV (**Figure 3H**), and CO (**Figure 3F**) were reduced 5 days after the induction of HUS, of which CO was also reduced 3 days after the induction of HUS. Taken together, the changes indicate intravascular volume depletion exacerbated by bradycardia.

### 3.7 The effect of a single high-dose challenge with Stx on plasma biomarkers of cardiac injury

**Figure 4:**
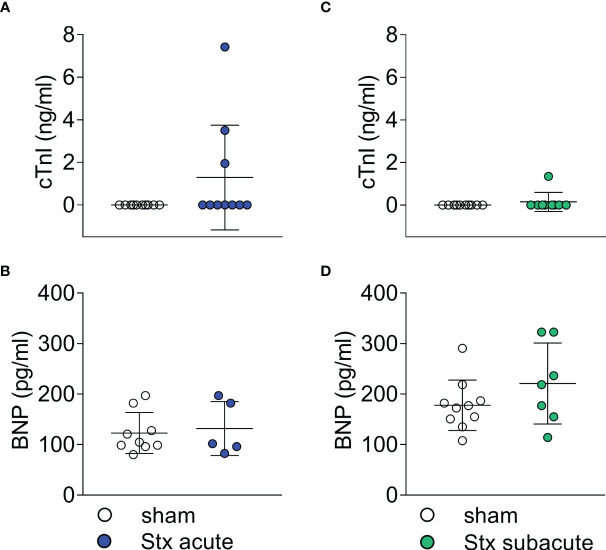
Plasma surrogate markers of cardiac injury in mice with hemolytic–uremic syndrome (HUS) exposed to different Shiga toxin (Stx) application regimes. **(A, B)** C57BL/6J mice received either a single high dose of 300 ng/kg Stx (Stx acute) or vehicle (sham). Blood was taken at the end of the experiment, 2 days after the induction of HUS. **(A)** Plasma concentrations of cardiac troponin I (cTnI) (sham *n* = 10, Stx acute *n* = 10) and **(B)** brain natriuretic peptide (BNP) (sham *n* = 9, Stx acute *n* = 5) were measured as surrogate parameters of cardiac injury. **(C, D)** Mice received either two lower doses of 25 ng/kg Stx spaced 72 h apart (Stx subacute) or vehicle (sham). Blood was taken at the end of the experiment 5 days after the induction of HUS **(C)** Plasma concentrations of cTnI (sham *n* = 10, Stx acute *n* = 9) and **(D)** BNP (sham *n* = 10, Stx subacute *n* = 7) were measured as surrogate parameters of cardiac injury. **(A–D)** Data are presented as scatter dot plot with mean ± SD, *p* < 0.05 vs. corresponding sham group (Mann–Whitney *U* test).

In plasma samples, we determined the concentration of cTnI and BNP as markers of cardiac injury in the acute model of HUS 2 days after the induction of HUS (**Figures 4A, B**). Three of 10 Stx-treated mice demonstrated increased cTnI levels (**Figure 4A**). All mice with increased cTnI levels exhibited clinical deterioration (HUS score = 3, **Supplementary Figure 1**); however, other mice with comparable disease severity regarding clinical presentation and laboratory values did not present with increased cTnI levels. The echocardiographic analysis revealed no apparent differences in this small subgroup compared with the other Stx-challenged mice. There were no differences in BNP levels between Stx- and sham-treated mice (**Figure 4B**).

### 3.8 The effect of repeated administrations of lower doses of Stx on plasma biomarkers of cardiac injury

In plasma samples, we determined the concentration of cTnI and BNP as markers of cardiac injury in the subacute model of HUS (**Figures 4C, D**) 5 days after the induction of HUS. cTnI was only detectable in one of the nine Stx-challenged mice (**Figure 4C**). This animal exhibited a high level of disease severity (HUS score = 3, **Supplementary Figure 1**), the highest NGAL level in plasma, and the lowest CO in this group. There were no differences in BNP levels between Stx- and sham-treated mice in this dose regimen (**Figure 4D**).

### 3.9 The effect of a single high-dose challenge with Stx on plasma electrolytes and electrocardiograms

**Figure 5:**
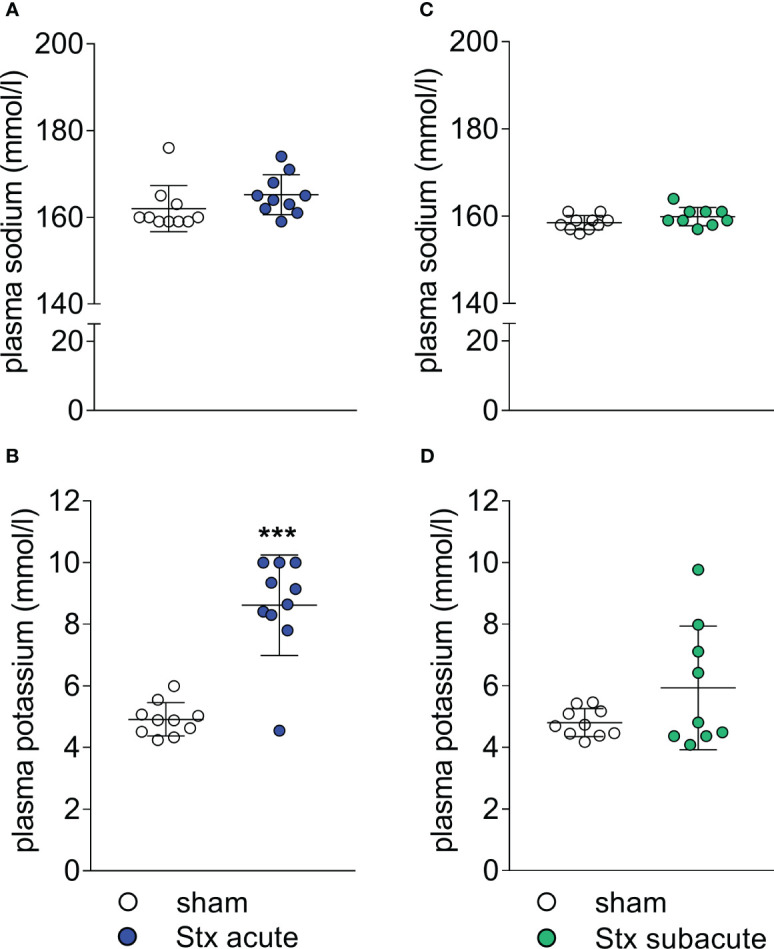
Plasma electrolyte concentrations in mice with hemolytic–uremic syndrome (HUS) exposed to different Shiga toxin (Stx) application regimes. **(A, B)** C57BL/6J mice received either a single high dose of 300 ng/kg Stx (Stx acute) or vehicle (sham). Blood was taken at the end of the experiment, 2 days after the induction of HUS. Plasma concentrations of **(A)** sodium and **(B)** potassium (sham *n* = 10, Stx *n* = 10). **(C, D)** Mice received either two lower doses of 25 ng/kg Stx spaced 72 h apart (Stx subacute) or vehicle (sham). Blood was taken at the end of the experiment, 5 days after the induction of HUS **(C)**. Plasma concentrations of **(C)** sodium and **(D)** potassium (sham *n* = 10, Stx *n* = 9). **(A–D)** Data are presented as scatter dot plot with mean ± SD, ***p < 0.001 vs. the corresponding sham group (Mann–Whitney *U* test).

We analyzed the concentrations of electrolytes in plasma samples of mice in the acute model of HUS 2 days after the induction of HUS (**Figures 5A, B**). When compared with sham-treated mice, plasma sodium concentrations were unchanged (**Figure 5A**). All but one Stx-treated mouse showed increased potassium levels (hyperkalemia, **Figure 5B**). The electrocardiograms that were recorded during echocardiography after reaching a steady state of anesthesia showed no morphological abnormalities in sham-treated mice (**Supplementary Figure 2**). All stx-treated mice with hyperkalemia showed widening of the QRS complexes and increased height of T waves. A subset of hyperkalemic mice showed bradycardia (**Supplementary Figure 3**).

### 3.10 The effect of repeated administrations of lower doses of Stx on plasma electrolytes and electrocardiograms

We analyzed the concentrations of electrolytes in plasma samples of mice in the subacute model of HUS 5 days after the induction of HUS (**Figures 5C, D**). When compared with sham-treated mice, plasma sodium concentrations showed no significant changes (**Figure 5C**). Four of nine plasma samples of Stx-treated mice showed hyperkalemia (**Figure 5D**). The electrocardiograms that were recorded during echocardiography after reaching a steady state of anesthesia showed no morphological abnormalities in sham-treated mice (**Supplementary Figure 4**). Two Stx-treated mice displayed bradycardia, widening of the QRS complexes, and increased height of T waves (**Supplementary Figure 5**), of which one had hyperkalemia and one lacked the plasma potassium value due to insufficient blood sample size.

## 4 Discussion

**Figure 6:**
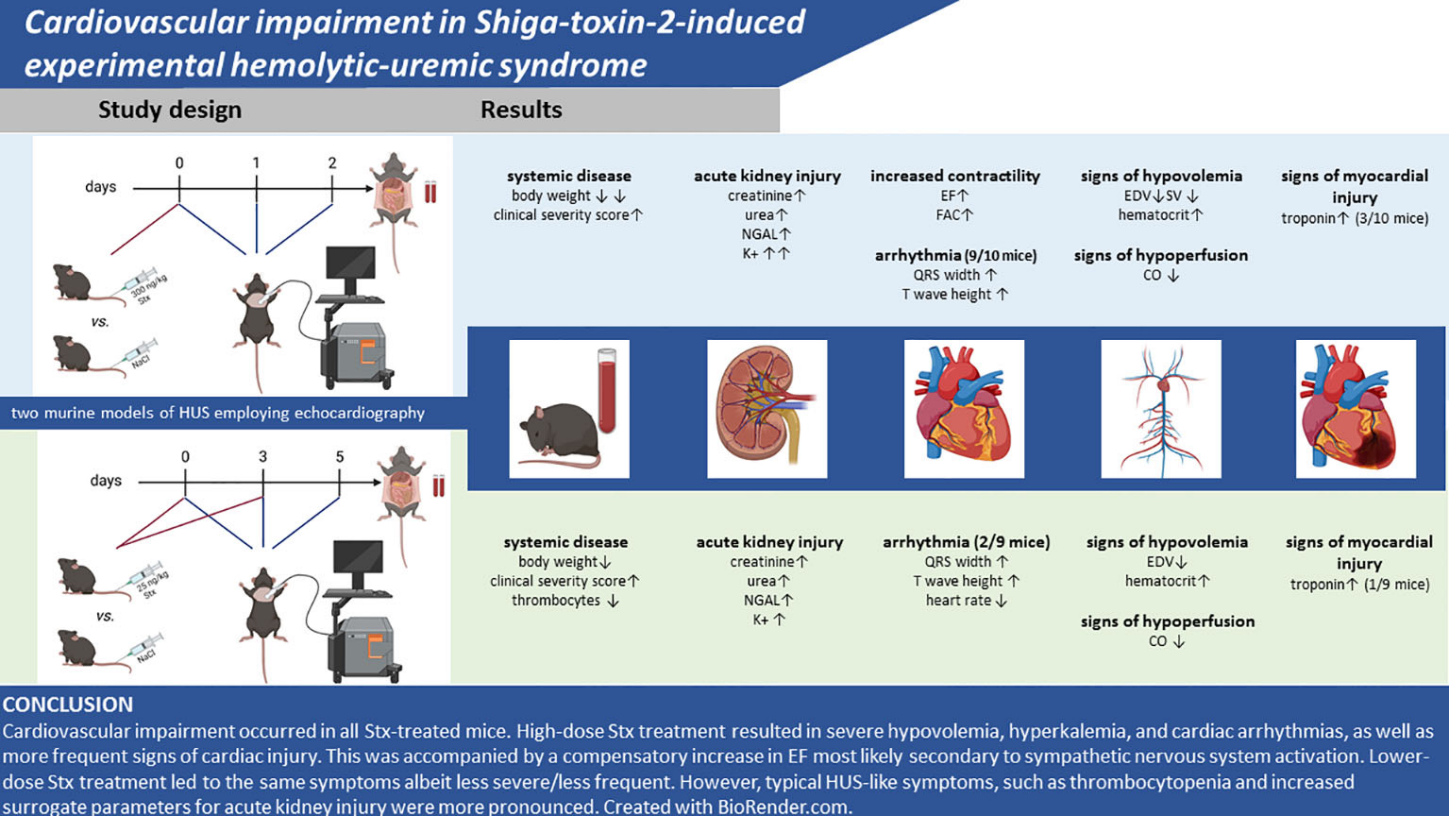
Summary of findings. Created with BioRender.com.

In this exploratory pilot study, we addressed and described for the first time Stx2-induced cardiovascular impairment in preclinical models of HUS. In two distinct experimental setups, we induced disease by either a single high or repeated lower doses of Stx to model an acute or subacute course of disease in mice and found results comparable with previous experiments in these models (16) regarding clinical and laboratory signs of disease, such as disease severity score, weight loss, and indicators of acute kidney injury. In addition to these previously reported readouts, to assess the effect of Stx on the cardiovascular system, echocardiographic and laboratory assessments of cardiac injury were performed, yielding signs of cardiovascular dysfunction with varying degrees in both Stx-treated groups. Both Stx dosage regimens resulted in an impairment of cardiovascular function as assessed by echocardiography. All of these novel findings are summarized in **Figure 6**.

### 4.1 Stx-induced hypovolemia contributes to cardiovascular impairment

Based on an observed hemoconcentration in previous experiments with Stx-induced HUS in mice, we hypothesized that the expected and well-documented hypovolemia may, despite volume substitution, be the cause of the observed cardiovascular dysfunction (which was particularly pronounced in the acute HUS model). We report here for the first time that both murine models of HUS caused by (two different regimens of) administration of Stx resulted in a reduction in CO, EDV, and SV. This was associated with reductions in heart rate in hyperkalemic mice, which, in turn, further exacerbated the deleterious effects of Stx on CO. In addition, hemoconcentration was observed in both models. Thus, the loss of intravascular volume (secondary to endothelial injury-dysfunction and plasma leak) results in a reduction in pre-load, which may have importantly contributed to the observed reductions in CO and SV in HUS mice. In the acute model of HUS, we found (in the early stages of the disease) an increase in EF and FAC, which may be reflective of a compensatory response to hypovolemia secondary to activation of the sympathetic nervous system. The increases may appear inconsistent with the observed decrease in CO. As EF is influenced by changes in both (or either) end-diastolic and end-systolic volumes, EF offers no reliable information on the absolute volume ejected from the heart (and, hence, SV or CO). This means that changes in EF are not necessarily mirrored by changes in CO. Interestingly, the increases in EF associated with decreases in CO observed in our murine model of HUS are also frequently observed in patients with sepsis. Notably, the cardiovascular impairment was less pronounced in the subacute model of HUS. Though often masked by hemolytic anemia, hemoconcentration secondary to dehydration is present in human HUS and has been identified as a risk factor for the development of neurological sequelae (17). Capillary leakage, which has been suspected in human HUS (18), may account for or aggravate intravascular hypovolemia. Despite hypovolemia, there was no increase in heart rate in Stx-treated mice compared with controls. This is consistent with findings from other murine models of hypovolemia: Vogel also found no difference in heart rates in hypovolemic compared with euvolemic BALB/c mice (19). In a more recent study, C57BL/6 did not react with an increase in heart rate in response to hemorrhage (20). In this experimental model of hemorrhagic shock, a mean increase in heart rate of 30–40 beats per minute was observed after fluid resuscitation, which is in the range of variance in our mouse model.

### 4.2 Stx induces cardiac injury in a subset of mice

A third of Stx-challenged mice in the acute model and one mouse in the subacute model showed increases of cTnI. This is likely secondary to cardiac injury (necrosis of cardiomyocytes). The disease severity in these specific mice was high (as judged by the clinical severity score), but other mice in the acute model with no measurable cTnI presented with even more severe disease. Furthermore, these mice showed no reduction in left-ventricular function or differences in the ST segment of the ECG (both if observed could reflect an acute myocardial infarction secondary to the obstruction of a coronary artery; see below) when compared to mice with normal troponin values. Hence, the role of cardiac injury (rise in cTnI levels) in the observed cardiac dysfunction and, indeed, overall outcome in this HUS model remains unclear. Increased troponin levels in pediatric patients with HUS have been described in both case reports and epidemiological studies (3, 6, 21, 22); however, in the absence of larger studies, the incidence of this laboratory finding in human HUS remains unknown. Therefore, the employed models mimic a potentially important part of HUS pathophysiology, previously often neglected. The origin of the increase in cTnI in HUS remains unknown; however, thrombotic microangiopathy of cardiac vessels (see above) has been found in two autopsy studies of children who died from HUS (23). In a case report of cardiovascular impairment in HUS, the temporal dynamics of plasma troponin were recorded around the cardiac event, showing a peak troponin level 24 h after the event and mild elevations 8 h before the event (5). Should the mouse model prove consistent with this finding, the mild increase in cTnI levels seen in some mice could potentially be indicative of a future cardiac event rather than a past one (i.e., acute myocardial infarction). It should be noted that any observed increases in cTnI levels may also be secondary to an impairment in renal function (7). This is particularly important given the presence of acute kidney injury and impaired renal function in both HUS models.

### 4.3 Arrhythmias secondary to hyperkalemia may be the cause of significant morbidity

In the analysis of plasma, we consistently observed hyperkalemia in a subgroup of Stx-treated mice. Hyperkalemia was found more frequently in mice receiving the higher dose of Stx, which may indicate a dose-dependent mechanism. AKI frequently causes electrolyte imbalances and the presence of hyperkalemia in this murine HUS model reflects findings from studies of human HUS. Hyperkalemia was found in 27.2% of a cohort of 33 children with HUS (24). Zhao et al. found hyperkalemia in 30% of 20 children with diarrhea-positive HUS in their clinical study (25). The frequency of hyperkalemia in humans is, therefore, similar to that seen in our subacute model of HUS. In our mouse model, we also observed electrocardiographic abnormalities in Stx-challenged mice; we found pathological ECGs typical of and exclusively in mice with hyperkalemia, suggesting hyperkalemia as the underlying cause. One case report describes ventricular arrhythmias following myocarditis and hyperkalemia in human HUS (26). In a case–control study of 17 fatal cases of HUS, hyperkalemia was among the causes of death (27). This is also reflected here, as mice with hyperkalemia had a high clinical HUS score.

However, in human HUS, the electrolyte disturbances and associated acid–base imbalance can potentially be treated with drugs or by various renal replacement procedures if diagnosed in a timely manner. If hyperkalemia is not treated, the resulting conduction disturbances may limit animal survival in preclinical models. Future preclinical models could include electrophysiological analyses of Stx-treated cardiomyocytes to investigate an inherent pro-arrhythmogenic effect of Stx.

### 4.4 Conclusion and outlook

Cardiovascular impairment occurred in all Stx-treated mice. High-dose Stx treatment resulted in severe hypovolemia, hyperkalemia, and cardiac arrhythmias, as well as more frequent signs of cardiac injury. This was accompanied by a compensatory increase in EF most likely secondary to sympathetic nervous system activation. Lower-dose Stx treatment led to the same symptoms albeit less severe/less frequently; however, typical HUS-like symptoms, such as thrombocytopenia and increased surrogate parameters for acute kidney injury, were more pronounced. This exploratory study is limited by its small sample size and lack of mechanistic investigation. The brevity of the model limits the analysis of certain parameters of heart failure such as heart weight/body weight ratio, which may also be skewed by the weight loss of the mice. It poses a first proof of principle, that both models are, in general, suitable models for the investigation of the pathophysiology of cardiovascular events in HUS, as both dose regimens present findings of cardiovascular impairment observed in human HUS. Further experiments should include histopathological analysis, in particular trichrome-stained images of the heart and markers of cardiac inflammation as a first investigation into the mechanisms underlying this impairment. It would be very useful to further gain a more detailed understanding of the hemodynamic alterations associated with both murine models of HUS reported here by characterizing the hemodynamics beyond this pilot study by continuous recording of blood pressure (e.g., an implantable device). This unfortunately was beyond the scope of this study. It is also not clear whether the surgical intervention necessary to implant the device that would allow a remote hemodynamic monitoring (first hit) would affect the subsequent response to Stx (second hit) and, hence, affect disease severity and pathology (even if not related to cardiac dysfunction). Future translational studies should consider the prognostic relevance of cardiovascular impairment, including hyperkalemia-induced arrhythmias, intravascular volume depletion, and decline of CO for the outcome and mortality of this rare but potentially fatal complication.

## Data availability statement

The raw data supporting the conclusions of this article will be made available by the authors, without undue reservation.

## Ethics statement

The animal study was approved by Thuringian State Office for Consumer Protection, Bad Langensalza, Germany (registration number 02 073/16). The study was conducted in accordance with the local legislation and institutional requirements.

## Author contributions

Conceptualization: SC; Methodology: SC; Validation: CN, BW, and SC; Formal analysis: CN and BW; Investigation: CN and BW; Resources: SC; Data curation: CN and BW; Writing—original draft preparation: CN, SC, BW, and CT; Writing—review and editing: CN, CT, and SC; Visualization: BW and CN; Supervision: SC; Project administration: SC; Funding acquisition: SC. All authors contributed to the article and approved the submitted version.
