# Supplementary material for: Cardiovascular impairment in Shiga-toxin-2-induced experimental hemolytic–uremic syndrome: a pilot study
Source: Front Immunol. 2023 Sep 22;14:1252818. doi: 10.3389/fimmu.2023.1252818 (PMC10556238; doi:10.3389/fimmu.2023.1252818)
Supplement: Supplementary file 1 [file DataSheet_1.pdf]

frontiers

Supplementary Material

Cardiovascular impairment in Shiga-toxin-2-induced experimental hemolytic-uremic syndrome

Charles Neu[1,2,3†], Bianka Wissuwa[1,2†], Christoph Thiemermann[4], Sina M. Coldewey[1,2,3]

[1]Department of Anesthesiology and Intensive Care Medicine, Jena University Hospital, Jena, Germany, [2]Septomics Research Center, Jena University Hospital, Jena, Germany, [3]Center for Sepsis Control and Care, Jena University Hospital, Jena, Germany, [4]William Harvey Research Institute, Barts and the London School of Medicine and Dentistry, Queen Mary University of London, London, United Kingdom

[†]These authors have contributed equally to this work

*** Correspondence:**

Prof. Sina M. Coldewey, MD, PhD,

Department of Anesthesiology and Intensive Care Medicine

Jena University Hospital,

Am Klinikum 1

07747 Jena, Germany

sina.coldewey@med.uni-jena.de (ORCID 0000-0002-7130-0006)

Phone: +49 3641 9 323101

Fax: +49 3641 9 323102

1 Supplementary Figures

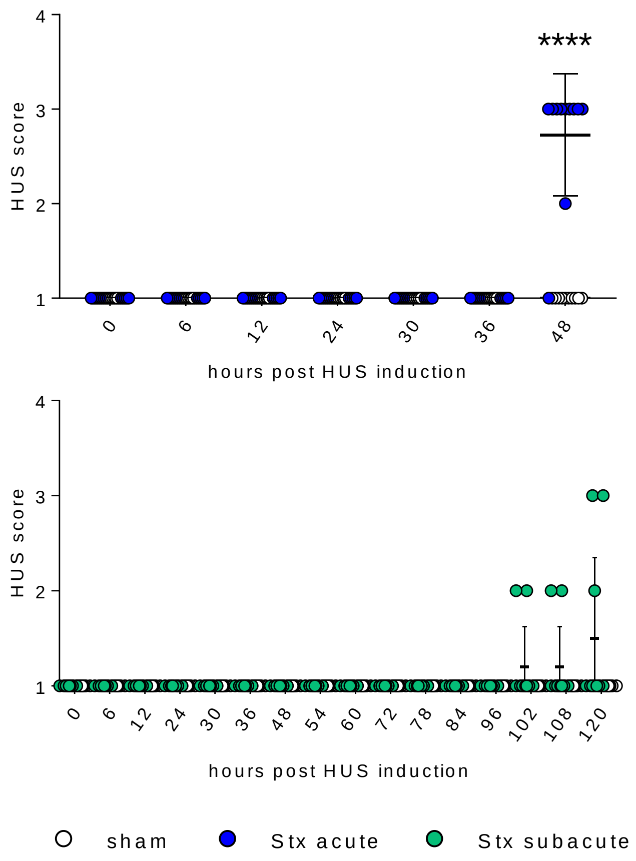

Supplementary Figure 1. Disease progression of C57BL/6J mice after induction of HUS.

HUS score in the acute (sham n=10, Stx n=11) and subacute model (sham n=10, Stx n=10). Data are presented as scatter dot plot with mean±SD. ****$P<0.0001$ vs. corresponding sham group (Mann-Whitney test for each respective time point).

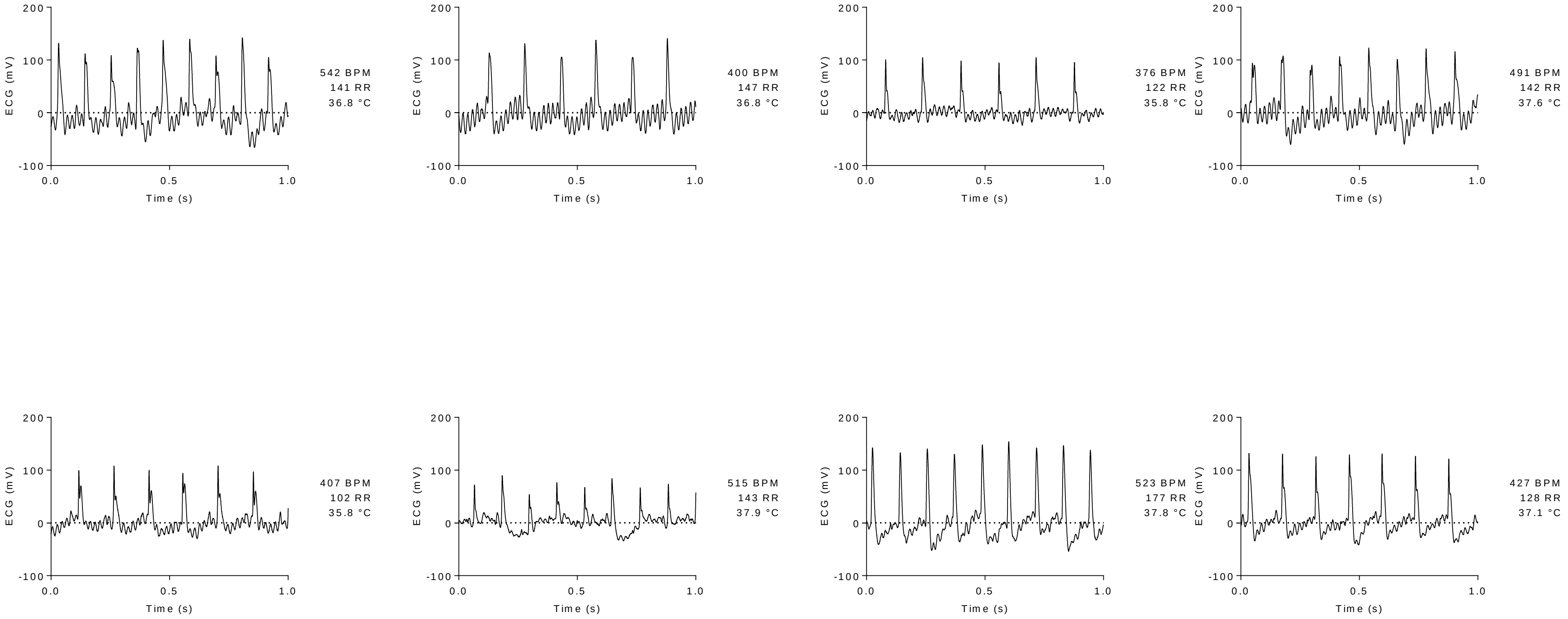

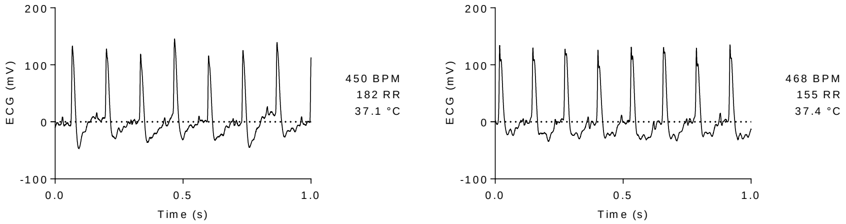

Supplementary Figure 2. Example electrocardiograms (ECGs) of sham-treated mice in the acute model of HUS

Each panel depicts one second of the electrocardiogram of an individual mouse. ECGs, heart rate in beats per minute (BPM), respiration rate (RR) and body temperature were recorded after reaching a steady state of anesthesia.

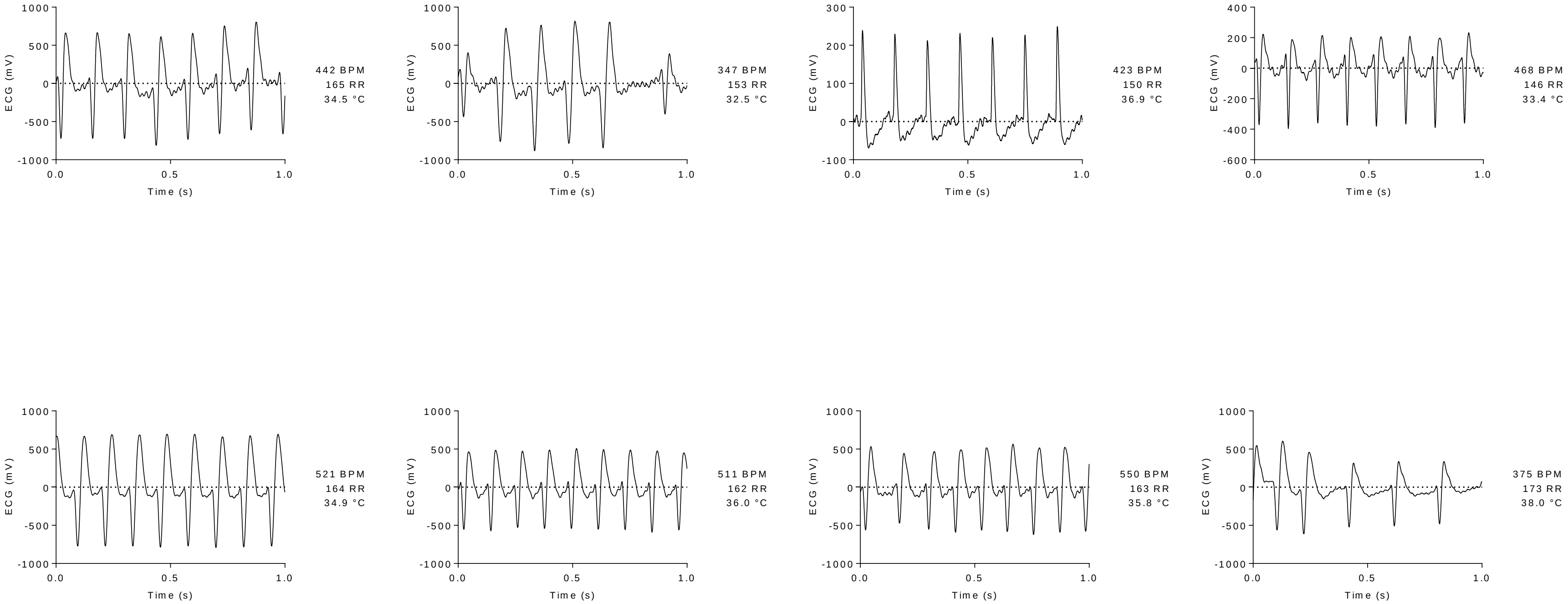

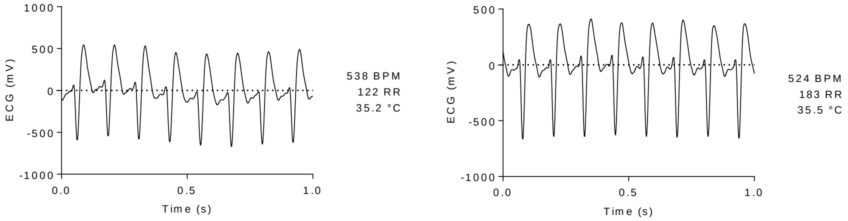

Supplementary Figure 3. Example electrocardiograms (ECGs) of Stx-treated mice in the acute model of HUS

Each panel depicts one second of the electrocardiogram of an individual mouse. ECGs, heart rate in beats per minute (BPM), respiration rate (RR) and body temperature were recorded after reaching a steady state of anesthesia.

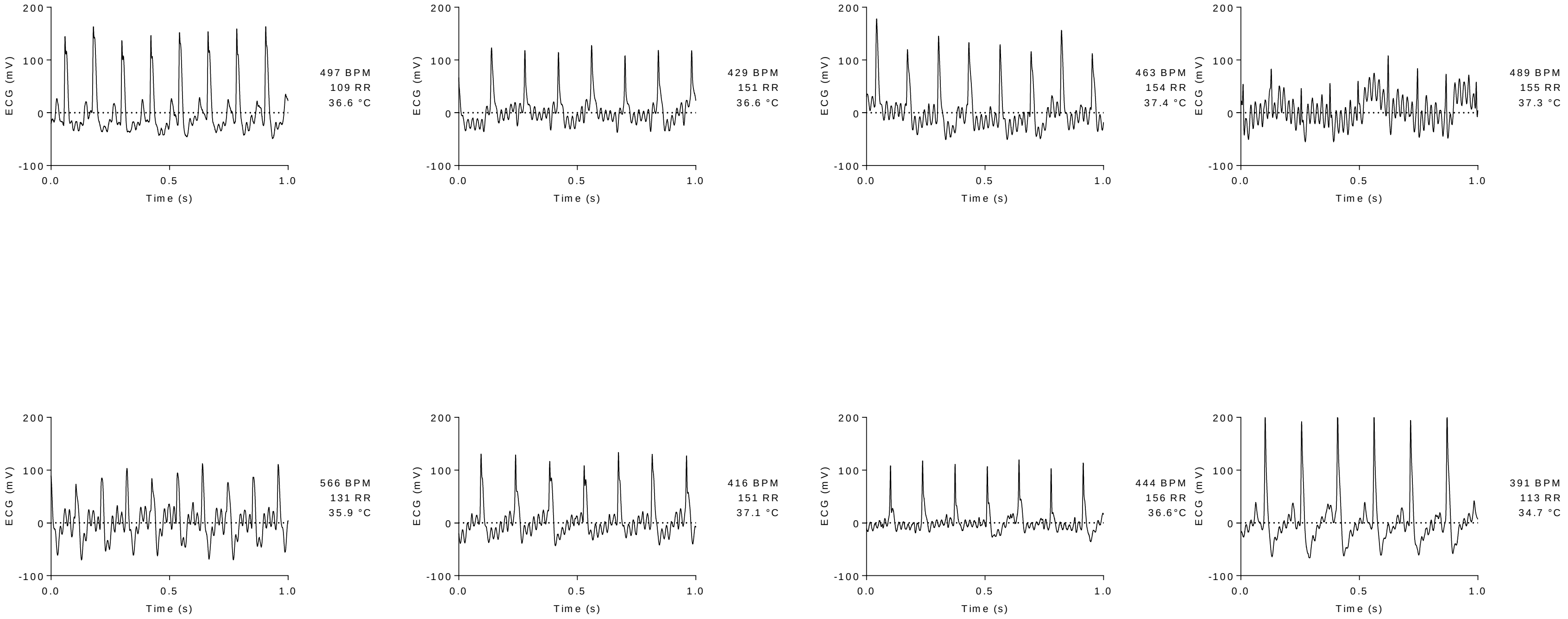

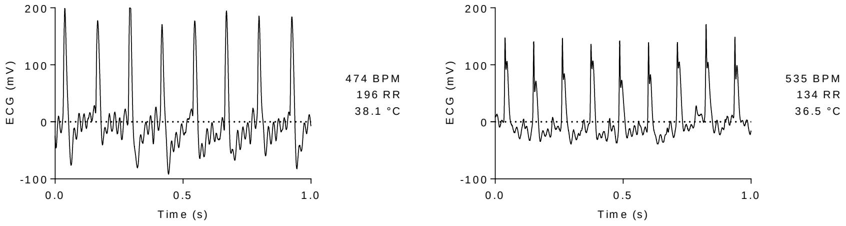

Supplementary Figure 4. Example electrocardiograms (ECGs) of sham-treated mice in the subacute model of HUS

Each panel depicts one second of the electrocardiogram of an individual mouse. ECGs, heart rate in beats per minute (BPM), respiration rate (RR) and body temperature were recorded after reaching a steady state of anesthesia.

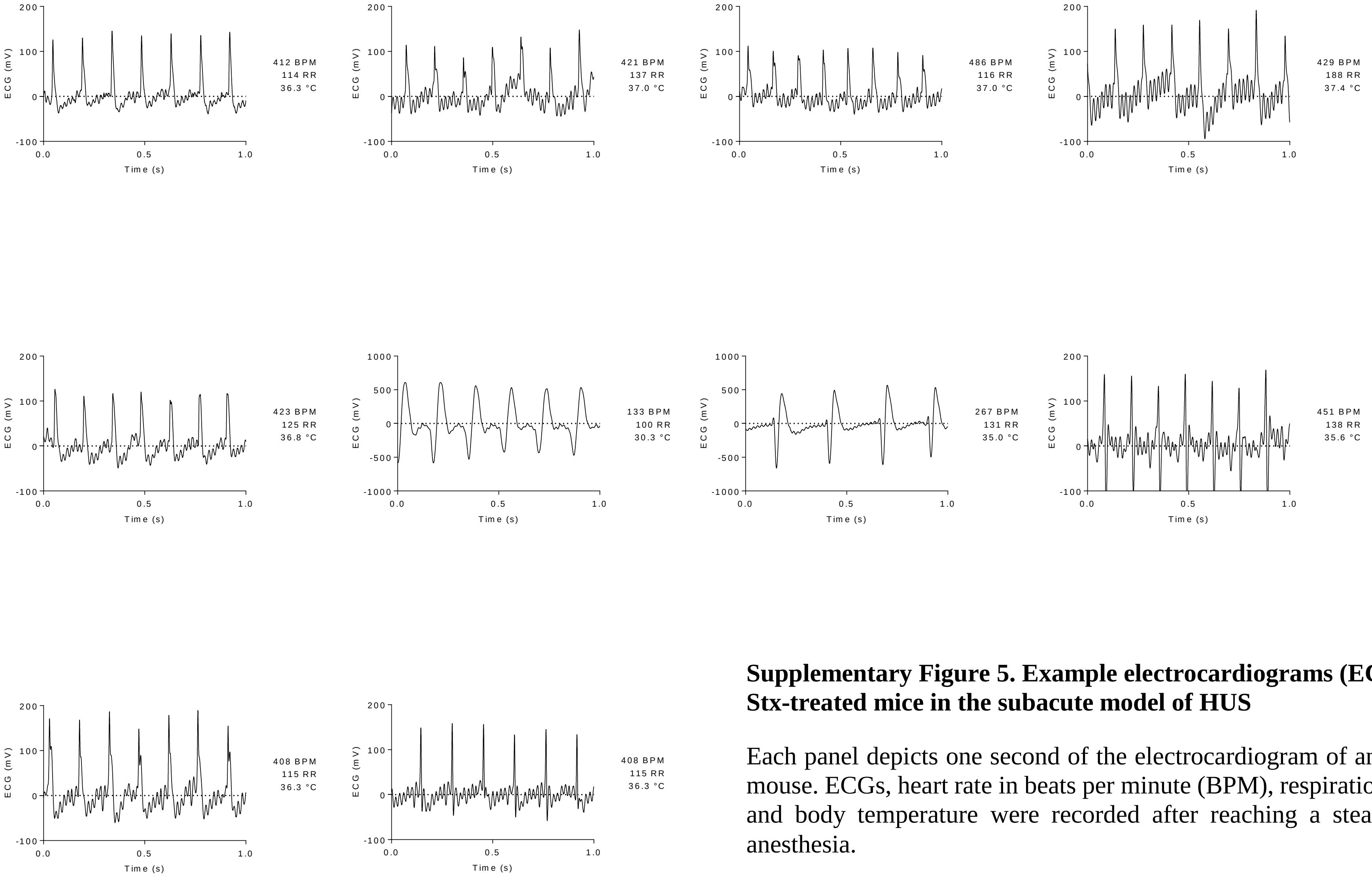

Supplementary Figure 5. Example electrocardiograms (ECGs) of Stx-treated mice in the subacute model of HUS

Each panel depicts one second of the electrocardiogram of an individual mouse. ECGs, heart rate in beats per minute (BPM), respiration rate (RR) and body temperature were recorded after reaching a steady state of anesthesia.
